# A Study on Machine Learning Methods’ Application for Dye Adsorption Prediction onto Agricultural Waste Activated Carbon

**DOI:** 10.3390/nano11102734

**Published:** 2021-10-15

**Authors:** Seyedehmaryam Moosavi, Otilia Manta, Yaser A. El-Badry, Enas E. Hussein, Zeinhom M. El-Bahy, Noor fariza Binti Mohd Fawzi, Jaunius Urbonavičius, Seyed Mohammad Hossein Moosavi

**Affiliations:** 1 Department of Chemistry and Bioengineering, Vilnius Gediminas Technical University, 10223 Vilnius, Lithuania; jaunius.urbonavicius@vilniustech.lt; 2 Romanian Academy, Center for Financial and Monetary Research “Victor Slavescu”, 050711 Bucharest, Romania; otilia.manta@rgic.ro; 3 Research Department, Romanian-American University, 012101 Bucharest, Romania; 4 Chemistry Department, Faculty of Science, Taif University, Khurma, P.O. Box 11099, Taif 21944, Saudi Arabia; y.elbadry@tu.edu.sa; 5 National Water Research Centre, P.O. Box 74, Shubra EI-Kheima 13411, Egypt; enas_el-sayed@nwrc.gov.eg; 6 Chemistry Department, Faculty of Science, Al-Azhar University, Cairo 11884, Egypt; zeinelbahy@azhar.edu.eg; 7 Nanotechnology & Catalysis Research Centre (NANOCAT), Institute for Advanced Studies (IAS), University for Malaya (UM), Kuala Lumpur 50603, Malaysia; farizafawzi@um.edu.my; 8 Faculty of Engineering, Centre for Transportation Research (CTR), University of Malaya (UM), Kuala Lumpur 50603, Malaysia; mh.moosavi65@gmail.com

**Keywords:** machine learning, wastewater treatment, dye adsorption, agricultural waste, activated carbon

## Abstract

The adsorption of dyes using 39 adsorbents (16 kinds of agro-wastes) were modeled using random forest (RF), decision tree (DT), and gradient boosting (GB) models based on 350 sets of adsorption experimental data. In addition, the correlation between variables and their importance was applied. After comprehensive feature selection analysis, five important variables were selected from nine variables. The RF with the highest accuracy (R^2^ = 0.9) was selected as the best model for prediction of adsorption capacity of agro-waste using the five selected variables. The results suggested that agro-waste characteristics (pore volume, surface area, agro-waste pH, and particle size) accounted for 50.7% contribution for adsorption efficiency. The pore volume and surface area are the most important influencing variables among the agro-waste characteristics, while the role of particle size was inconspicuous. The accurate ability of the developed models’ prediction could significantly reduce experimental screening efforts, such as predicting the dye removal efficiency of agro-waste activated carbon according to agro-waste characteristics. The relative importance of variables could provide a right direction for better treatments of dyes in the real wastewater.

## 1. Introduction

Approximately 10,000 dyes are commercially available, and annually, about 1.6 million tons of dyes are produced for industrial use [1], of which 10–15% of these dyes are disposed of as wastewater [2]. This pollution is caused by the use of dyes in the clothing, paper, dyeing, and plastics industries. Because the dyes are very stable and solvable in water, failed dye treatment and disposal of these wastes into receiving waters causes huge damages to the environment: affecting photosynthetic activity [3]; being toxic to aquatic life due to the presence of metals, chlorides, etc. [4]; and inherent toxicity, mutagenicity, and carcinogenicity [5]. In addition, overexposure to dyes has resulted in potentially life threatening complications such as skin harms, respiratory problems, and the probability of human carcinoma [6].

Most of these synthetic dyes are chemically and thermally stable, non-biodegradable, and quite toxic [7]. Due to toxicity of dyes, it is necessary to remove them from wastewater before them discharge to the natural environment. Adsorption is generally considered to be the most prominent approach due to its effectiveness [8], economy, and simplicity for quickly lowering the concentration of dissolved dyes in an effluent, as it does not require a pretreatment step before its application [9]. This method is very dependent upon the type of the adsorbent used (e.g., activated carbon (AC), biomass, polymer, nanomaterial, etc.) [10]. The cost of adsorbent production is not the only factor involved in developing an excellent adsorbent. The adsorption performance, regeneration ability, and adsorbent separation are other important features of an effective adsorbent [11].

Among the large number of adsorbents, activated carbons (ACs) exhibited advantages over other adsorbents for their high surface area, microporous character, chemical nature of their surface, and high adsorption capacity when used on wastewater with different dye molecules [12]. Most of the activated carbon materials used for adsorption research come from fruit peel [13], rubber tires [14], textile sludge [15], crab shell [16], and egg shell [17]. Recently, agricultural wastes have received considerable attention due to their abundant surface functional groups, porous structures, additional inorganic minerals, and high surface area. Many researchers study the dyes’ adsorption on various agro-waste materials such as corn cobs [18], oil cake [19], rice husk [20], sugarcane bagasse [21], gram husk [22], sawdust [23], pine cone [24], tobacco residue [25], white sugar [26], Astragalus bisulcatus tree [27], tea residue [28], vinasse [29], groundnut shell [30], and so on. This class of activated carbons have a valuable potential for the wastewater dye removal. Agro-waste ability in the treatment of wastewater containing dyes such as MB dyes could reach the maximum value of 2251 mg/g [29]. Most of current research followed similar methods for AC for preparation under appropriate pyrolysis temperature, followed by measuring the removal value of any types of dyes at different environmental conditions like solution pH and initial dye concentration. The adsorption kinetic, isotherm, and maximum dye removal value were subsequently modelled and confirmed based on the obtained data. The adsorption capacity of adsorbate onto the adsorbent is determined at ambient condition (under certain experimental conditions). The procedure to obtain relative contributions of adsorption mechanisms (like initial concentration) using different agro-waste AC characteristic (like surface area, pore volume, pH, and particle size) were time consuming and complex. In the above, holistic adsorption mechanisms were not considered. In order to better deal with dyes in real water and wastewater, it is essential to understand the relative importance of each variable so as to gain the right solution for improving the adsorption capacity.

In recent years, machine learning (ML), playing an important role in computer science, artificial intelligence, chemistry, and biomedicine, has attracted people’s attention [31]. Unlike empirical models, such as the Freundlich and Langmuir models used to detect the adsorption equilibrium, which can barely predict conclusions and make the relationship between operating conditions and adsorption capacity unavailable [26]; today, the machine learning (ML) method is preferred through modeling and learning the behavior of adsorption on agro-waste [32]. It may be preferred to resolve the problem through modeling and learning the adsorption behavior of dyes onto agro-wastes. High-quality machine learning models used to predict the adsorption efficiency have the ability to reduce the complexity, numbers, and time of experiments and to find a non-linear mathematical relationship between dependent and independent input variables. In recent studies, developing ML models could remarkably decrease the material resources and amount of manpower for future experiments and research. The emerging machine learning models, especially the decision tree (DT), random forest (RF), and gradient boosting models, have been successfully proved to have the merit to model and predict complex and non-linear mathematical relationships between dependent and independent factors. Therefore, the three machine learning models were selected and compared to (i) develop models for predicting the efficiency of dye adsorption onto the agro-wastes AC, based on the agro-waste characteristics, initial concentration, type of dyes, and conditions of adsorption; (ii) evaluate and select the relative importance of each variables and select the most important variables; and (iii) predict dyes adsorption efficiency and find the intrinsic information behind the models based on the agro-waste characteristics, which is valuable for reducing unnecessary repetitive experiments.

The models developed in this study are used to predict dye adsorption efficiency in wastewater based on measurable agro-wastes AC characteristics such as surface area, pH, pore volume, and particle size. This study with the aid of machine learning, which would be valuable for future applications with the increasing accumulation of big data in the scientific literature, while detecting the relative importance of each factor in improving adsorption efficiency; it provides a comprehensive understanding of dye removal using agro-wastes and proposed guidelines for the treatment of wastewater and contaminated water containing dyes.

## 2. Materials and Methods

### 2.1. Data Collection

The dye adsorption experimental data on the agro-waste AC were obtained from previous studies. The adsorption value data were collected directly from the tables or extracted from the Supplementary Material data and graphs with Getdata 2.21 in the published papers. Ultimately, 350 sets of adsorption experimental data of different dyes on the agro-waste AC were selected [15,16,22,25,26,30,33,34,35,36,37,38,39]. Some of data were missing and needed to be deleted, because dealing with missing data could cause errors in our whole dataset. The detailed data were shown in the Table S1 in Supplementary Materials data file. All the 39 adsorbents were produced all from agro-wastes (16 kinds of agro-wastes) at the temperature range of 400–800 °C. The characteristics of the agro-wastes were varied due to different feedstock and pyrolysis conditions, where the statistical distributions of the factors related to the agro-waste characteristics were acquired via boxplots.

In this study, the adsorption process was modelled and the dyes’ adsorption capacity onto the agro-waste AC was predicted. Ten variables were considered as influencing factors and divided into four parts: (i) agro-waste characteristics, including surface area (SA, m^2^/g), agro-waste pH in water (pHH_2_O), pore volume (PV, cm^3^/g), and particle size (PS, mm); (ii) adsorption conditions, including adsorption temperature (T, °C) and pH of the solution (pHsol); (iii) dye initial concentration (C_0_, mg/g); and (iv) type of dyes. These explainable variables and adsorption efficiency values are summarized in Table S1.

### 2.2. Data Pre-Processing (Pre-Processing) and Model Estimators

The data processing algorithm was developed using the SK-learn module in Python 3.6 (Python Software Foundation, Beaverton, OR, USA). To eliminate the measurement unit, the predictors were normalized by mean and standard deviation before fitting in regression. In this paper, three methods were mainly used to compare model performance, gradient boosting (GB), decision tree (DT), and random forest (RF) models. To evaluate the accuracy of the model, the correlation coefficient (R), the mean squared error (MSE), and the root mean squared error were used.

(1) $$R=\sqrt{1-\frac{\sum_{i=1}^{N}{\left({\hat{y}}_{i}-y_{i}\right)}^{2}}{\sum_{i-1}^{N}{\left({\hat{y}}_{i}-\bar{y}\right)}^{2}}}$$

(2) $$MSE=\frac{1}{N}\overset{N}{\underset{i=1}{\sum }}{\left({\hat{y}}_{i}-\bar{y}\right)}^{2\;}\;\;$$

(3) $$RMSE=\sqrt{MSE}$$

The Pearson correlation coefficient (PCC) measured the linear dependences between any two selected variables or between each feature and the target variable, which were calculated with Equation (4):

(4) $$r_{xy}=\frac{\sum_{i=1}^{n}\left(x_{i}-\bar{x}\right)\sum_{i=1}^{n}\left(y_{i}-\bar{y}\right)}{\sqrt{\sum_{i=1}^{n}{\left(x_{i}-\bar{x}\right)}^{2}}\sqrt{\sum_{i=1}^{n}{\left(y_{i}-\bar{y}\right)}^{2}}}$$

where $\bar{x\;}$ or $\bar{y}$ = mean of factors x or y, respectively. Then, each variable’s data were normalized into the range of 0 and 1 with Equation (5):

(5) $$y=\frac{\left(x_{i}-x_{min}\right)}{\left(x_{max}-x_{min}\right)}$$

where *y* = normalized value of initial *x_i_*,and *x_max_* and *x_min_* = maximum and minimum value of *x_i_*, respectively.

### 2.3. Models Built with Decision Tree, Gradient Boosting, and Random Forest Methods

Decision tree classifiers (DTCs) are used successfully in many diverse areas such as radar signal classification, character recognition, remote sensing, medical diagnosis, expert systems, and speech recognition, to name only a few. Perhaps the most important feature of DTCs is their capability to break down a complex decision-making process into a collection of simpler decisions, thus providing a solution that is often easier to interpret [40]. Decision trees classify inputs into branch-like segments by taking paths from the root node (implies the prediction or that gives the best split of the target class values) through internal nodes to leaf nodes [41]. Each internal node contains splits and holds two or more child nodes, and further, splitting is applied iteratively to the subgroups until leaf nodes are obtained. The decision tree process is completed in one of these aspects: (a) the class label of the leaf node is the same as the target class value, (b) every prediction is used to split a partition, and (c) there are no more records for a particular value of a prediction or variable.

The training process is used to build and evaluate the decision tree model by minimizing the difference between the measured and predicted outputs. These procedures are applied to estimate the accuracy of the decision tree by comparing predicted outputs with actual data. Finally, a decision tree can be employed for classification and prediction purposes using a new dataset [41].

The random forest (RF) model, which works on the basis of bootstrap aggregation and multiple decision trees, is known as a supervised ensemble ML technique. It is included in the scope of the ensemble learning field. RF is in fact a bagging algorithm-based additive model. It should be noted that unlike bagging, RF builds each tree with the use of a random sample predictor before each node segmentation. This strategy results in a significant reduction in bias. The RF algorithm is a technique of classification, which makes use of a CART decision tree as the base classifier. In this model, every decision tree is produced in parallel, which could be either a regression tree or a classification tree. Each node within a decision tree is divided with the help of the optimal features capable of producing the optimum solution amongst all the available features. RF has been widely used in the literature in order to extract valuable, but there is hidden information in large volumes of data. This algorithm produces the training sets first with the application of the bootstrap method; afterward, it constructs a decision tree for each training set.

Each training subset was used to train various classifiers of the same type. After that, simple majority voting was applied to combining the individual classifiers. RF was implemented through three steps as follows: (1) performing the sampling process in a random way through dividing datasets into a number of subsamples; (2) training the decision trees with various subsamples, where each tree grew to the maximum degree on the basis of a bootstrap replicate of the training data, and each leaf node resulted the mean of all label values in the node; and (3) obtaining the final prediction through averaging the performance of all trees. To obtain the most desired model, the trial-and-error approach was used to optimize a number of tuning hyper-parameters, e.g., the number of trees (Ntree), the maximum number of features at each node (Nfeature), and the other input parameters. For the N case of each input variable, the relative importance was measured by means of the mean decrease impurity method. In this method, one can calculate how much each feature reduces the weighted impurity in a tree, and then they can average the reduction in the impurity from each feature and rank it for the developed forest.

Gradient boosting (GB) work based on the boosting principle, that is, to combine models with low variance error and high bias in a way to decrease bias and, at the same time, keep a low level of variance. Boosting learns multiple classifiers through altering the samples weight in the course of each training process. Then, it linearly integrates these classifiers with the aim of improving the classification performance. More specifically, boosting trees does not employ deep trees and various training datasets, rather, they prefer to make use of shallow trees trained in the same dataset; in this system, each tree is specialized in a definite characteristic of the relation between the input and output. In other words, succeeding shallow trees are trained in series, where the nth tree is trained in order to decrease the estimation errors of the former (*n* − 1)th trees.

GB is essentially aimed at developing an additive model to minimize the loss function. First, at the initialization step, GB starts with a constant value minimizing the loss function. After that, in each iterative training process, the negative gradient of the loss function is predicted as the residual value in the current model, and then a novel regression tree is trained in a way to be fitting the current residual. Then, the final step involves the addition of the current regression tree to the former model and updating the residual. The algorithm’s operation is continued iteratively until the stopping criterion (reaching the maximum number of iterations) is met. GB has been successful in improving the former poor performance of data through persistently employing the regression tree for the purpose of fitting the residuals. In the following, the GB algorithm is described briefly.

### 2.4. Statistical Evaluations

To evaluate the accuracy of the model, the correlation coefficient (R) and the root mean squared error (RMSE) were used.

(6) $$R^{2}=1-\frac{\sum_{i=1}^{N}{\left(Y_{i}^{exp}-Y_{i}^{pred}\right)}^{2}}{\sum_{i=1}^{N}{\left(Y_{i}^{exp}-{\bar{Y}}_{ave}^{exp}\right)}^{2}}$$

(7) $$RMSE=\sqrt{\frac{1}{N}\sum_{i=1}^{N}{\left(Y_{i}^{exp}-Y_{i}^{pred}\right)}^{2}}$$

where $Y_{i}^{exp}$ and $Y_{i}^{pred}$ = experimental value and predicted values, respectively, and ${\bar{Y}}_{ave}^{exp}$ = average of the experimental value.

## 3. Results and Discussion

### Comparison of the Models

**Figure 1:**
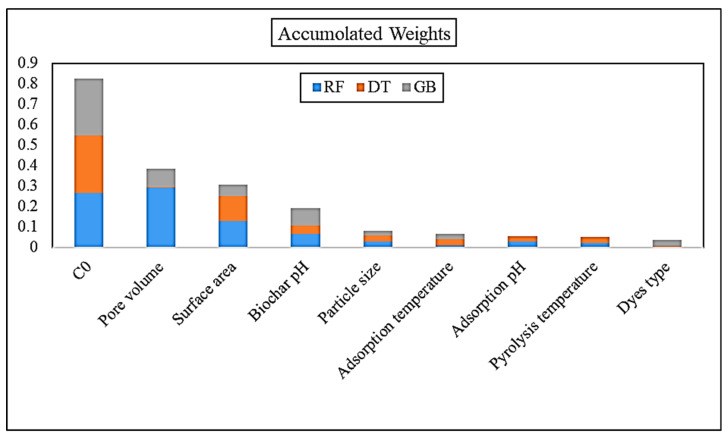
Accumulated weights of variables based on three ML methods.

Table 1 presents the nine independent variables’ weight based on output of the DT, RF, and GB techniques. According to this table, some mutual important variables were detected (for example, adsorption capacity, agro-waste pH, and surface area with highest weights in all three methods). The importance rank of the nine independent variables based on the DT, RF, and GB techniques is shown (in different colors) and presented in Figure 1. In this study, we adopted different feature selection methods to select only the important variables and develop our model based on selected variables. The main reason behind reducing the number of variables (based on their level of importance and correlations) is to reduce the complexity and improve the applicability of the final model. As shown in Figure 1, the weight values of each variable were summed up and compared. The accumulated weight values of the nine variables were sorted out from highest to lowest values in Table 1. As Table 1 presents, the initial concentration, surface area, and pore volume were detected as the most important variables, which is confirmed by previous studies. Therefore, the variables’ importance is used based on three different tree-based supervised machine learning techniques and the five most important variables according to the sum of three models are selected. Table 2 presented the variable importance of the five selected variables based on feature selection criteria.

The modeling using RF, GB, and DT was performed using nine variables (Table 3) and five selected variables (Table 4). According to Table 3, the R^2^ has a maximum value of 0.92 using the RF models and minimum R^2^ using the GB model. In addition, the RMSE and error have the lowest values using the RF models. Therefore, the overall RMSE value and R^2^ value developed by the RF algorithm were acceptable and more accurate compared to the other two models. The prediction ability of RF model for the pollutant adsorption onto the agro-waste was proved in another study [42].

In this study, the ML methods including RF, DT, and GB were used and discussed to determine the deep relationship between adsorption capacity and five selected influencing variables. Table 4 presents the modeling performance with the five variables. According to Table 4, RF had highest accuracy (R^2^ = 0.90) and lowest RMSE (0.0148) and absolute error (0.092) among the other two models. As it was expected, with decreasing the number of variables using feature selection, the accuracy decreased but only by 0.02. The modeling performance using the five variables was more valuable as we could reduce the complexity, and 2% difference is not much difference. Therefore, RF was competent and chosen as the most accurate model.

According to the Pearson correlation matrix (Table 5), the adsorption capacity was found to be the adsorption efficiency positively correlated with initial concentration, surface area, pore volume, and particle size and negatively correlated with the agro-waste pH. Some internal connections between the agro-waste characteristics were also detected with the Pearson correlation coefficient (PCC): (1) The particle size of agro-waste showed positive correlation with surface area and pore volume. This is because finer particles have a larger external surface exposed to heat, resulting in an extensive pore widening; thus, the surface area and micropore volume decreased as the latter transformed into mesopores [43]. (2) The agro-waste pH had an inverse correlation with pore volume and surface area. According to the previous studies, the higher carbonization degree meant removal of volatile matter agro-waste (has direct influence on carbon and nitrogen dynamics in soil) while higher ash percentage could reduce the surface area by filling micropores [44]. Moreover, ash content plays a main role in pH [45]. Therefore, the surface area of agro-waste showed a negative relation with the agro-waste pH (Table 5). In order to determine the deep relationship between the adsorption efficiency and these influencing factors, ML methods were used in the study and will be discussed in the next sections.

The Random Forest model was performed using the five selected variables and the importance of the variables is presented in Table 6. According to this table, the adsorption efficiency is highly affected by initial concentration (0.347), surface area (0.287), and pore volume (0.131), which is in line with previous findings [46,47]. The initial dye concentration has the highest effect on the adsorption capacity [47,48]. The increase of dye concentration gradients between the aqueous solution and adsorbent surface facilitated the adsorption of dye onto the agro-waste. Ins addition, the contribution of the main agro-waste characteristics was furtherly assessed in detail. It was found that pore volume was the main factor of agro-waste over the other properties of agro-waste. According to Table 6, the surface area is the second important factor of agro-wastee. A higher surface area could afford more active sites and an enhanced carbon/substrate interaction to improve the adsorption efficiency [49,50,51]. The impact of surface area on the adsorption capacity continuously increased within the range below ~600 m^2^/g, but a further increase in the range of 600–1700 m^2^/g showed a restrained trend, and over 1700 m^2^/g there was a sharp decrease in adsorption efficiency. A very large surface area (over 1700 m^2^/g) might have a negative impact on the other physicochemical characteristics of agro-waste like limited micropore accessibility, slow mass transfer, and diminished surface functional groups, which reciprocally influence the overall adsorption performance [39,52,53]. The evaluation of results in this study can contribute to present the adsorption of relevant water pollutants as dyes on adsorbents with high surface areas (like agro-waste).

**Figure 2:**
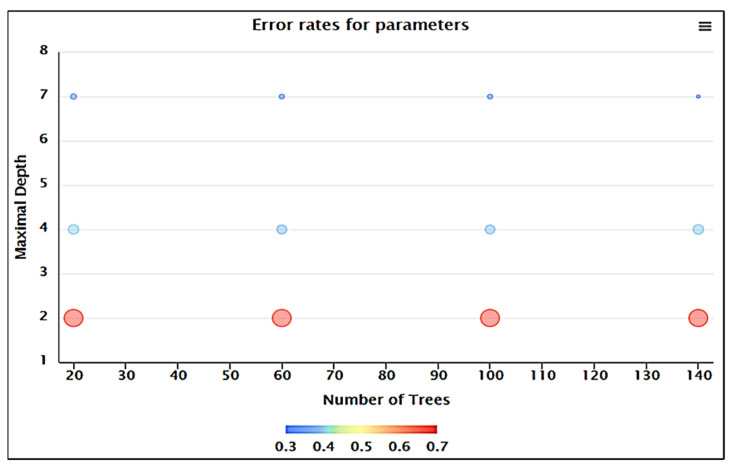
3D optimization process of RF models with error rates.

Additionally, the random forest (RF) model was optimized by simultaneously adjusting N_tree_ ranging from 20 to 140 and the maximal depth from 2 to 7 with a step size of 1 (Table 7). Model assessment was repeated 12 times using a different number of trees from 20 to 140, and the results are shown in Table 7. The minimum value of error rate was acquired when the N_tree_ and maximal depth were set as 140 and 7, respectively, which was easy to understand because the RF performance generally ameliorates with maximal depth increment due to higher number of available features to consider [54]. However, the best optimal model was acquired with the N_tree_ of 20 and maximal depth of 7 (no. 9 in Table 7). As shown in Table 7, the lowest error rate belonged to optimal model no. 12 (error rate = 0.347), which is very close to the error rate of model no. 9 (error rate = 0.351). Optimal model no. 9 was selected as the best model, because of it had the lowest N_trees_ (20) in comparison with N_trees_ of 140 (No. 12), and the error rate difference was only 0.004. The N_tree_ = 20 and maximal depth = 7 was used to assess the lowest error rate, as shown in Figure 2.

**Figure 3:**
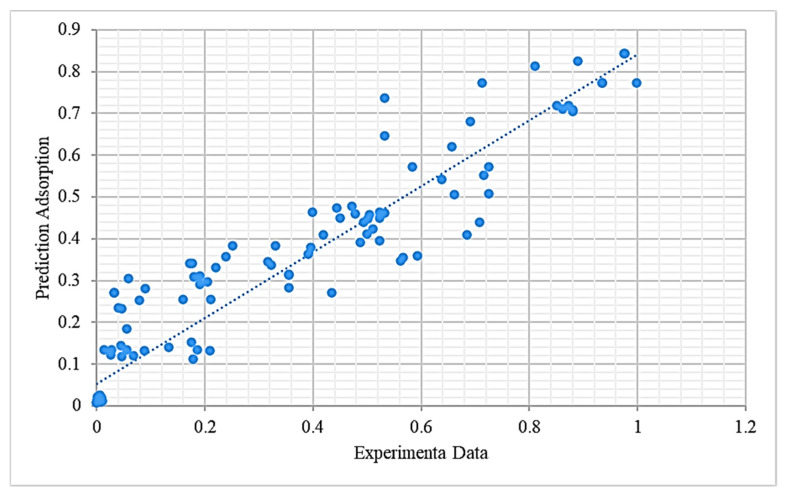
Predicted adsorption efficiency versus experimental data using random forest (RF) model.

The predicted results in the test groups plotted versus the corresponding experimental data with the RF models are presented in Figure 3. The blue line represented the regression line. As it can be seen in Figure 3, the predicted outputs were satisfactory and the RF model could present valuable overall predicted ability (R^2^ = 0.90).

For us, the accurate ability of the models’ prediction was valuable, but the underlying models’ information was more important based on such accurate predictions. For example, pore volume, surface area, and pH of agro-waste were illustrated as the most efficient variables for dyes’ adsorption, so this class of agro-wastes are quite appropriate to be used for wastewater treatment with dyes. For the target pollutants (dyes), the selected model narrowed down the target of searching to get the best agro-waste AC adsorbents and decreased the unnecessary attempts with adsorption experiments. Additionally, this could decrease the potential harm for environmental systems and researchers in doing routine experiments. In addition, the RF, DT, and GB only took 3, 8, and 32 s for one job in the study, respectively, which could significantly accelerate the research and applications of dye adsorption onto the agro-waste. Table 8 showed the optimized value of the selected variables’ values.

## 4. Conclusions

This study modelled the dye adsorption capacity onto agro-waste activated carbon using a machine learning approach based on nine variables: agro-waste characteristics, including surface area, agro-waste pH in water, pore volume, and particle size; (ii) adsorption conditions, including the adsorption temperature and pH of the solution; (iii) dye initial concentration; and (iv) type of dyes. RF, GB, and DT models were performed with a high accuracy of 0.92, 0.84, and 0.83, respectively. Five variables (initial concentration, pore volume, surface area, agro-waste pH, and particle size) were selected as the most effective variables on adsorption capacity. The three models were performed again on the five selected variables. The RF model with the highest accuracy (R^2^ = 0.9) was selected as the best model for the prediction of adsorption capacity on agro-waste AC. The results showed that agro-waste characteristics (pore volume, surface area, agro-waste pH, and particle size) accounted for a 50.7% contribution for adsorption capacity. The pore volume and surface area are the most effective variables among the agro-waste characteristics, while the role of particle size was inconspicuous. The accurate prediction for adsorption efficiency of dyes and the intrinsic information behind the models based on the agro-waste characteristics was valuable to reducing unnecessary repetitive experiments and the rational design and optimal selection of agro-waste material for dye removal from industrial wastewater with minimal experimental screening efforts.

## 5. Future Perspectives

The future research suggestions of ML models in the field of dye adsorption for carrying out extensive studies are as follows:

- The dye adsorption capability using other ML models such as the group method of data handling (GMDH).
- The application of machine learning models on dye adsorption using raw bio-waste.
- Specific study on the capability of ML models on the adsorption of pollutants like BOD and COD.

## Figures and Tables

**Table 1:** Importance score (weight) of variables based on three machine learning (ML) methods.

DT		RF		GB	
Attribute	Weight	Attribute	Weight	Attribute	Weight
C_0_	0.281	Pore volume	0.291	C_0_	0.279
Surface area	0.122	C_0_	0.267	Pore volume	0.086
Agro-waste pH	0.042	Surface area	0.131	Agro-waste pH	0.082
Pyrolysis temperature	0.03	Agro-waste pH	0.067	Surface area	0.055
Particle size	0.029	Adsorption pH	0.031	Dye type	0.032
Adsorption temperature	0.027	Particle size	0.029	Adsorption temperature	0.026
Adsorption pH	0.025	Pyrolysis temperature	0.022	Particle size	0.025
Pore volume	0.007	Adsorption temperature	0.013	Pyrolysis temperature	0
Dye type	0.004	Dye type	0.002	Adsorption pH	0

**Table 2:** Variable importance of the five selected variables based on the feature selection criteria.

Attribute	RF	DT	GB
C_0_	0.267	0.281	0.279
Pore volume	0.291	0.007	0.086
Surface area	0.131	0.122	0.055
Biochar pH	0.067	0.042	0.082
Particle size	0.029	0.029	0.025

**Table 3:** Modeling performance with nine variables.

	Train			Test		
Index	R^2^	RMSE	Absolute Error	R^2^	RMSE	Absolute Error
RF	0.92	0.116	0.076	0.84	0.127	0.098
G.B	0.84	0.162	0.076	0.76	0.081	0.100
D.T	0.83	0.178	0.098	0.71	0.130	0.167

**Table 4:** Modeling performance with five variables.

	Train			Test		
Index	R^2^	RMSE	Absolute Error	R^2^	RMSE	Absolute Error
RF	0.90	0.148	0.092	0.81	0.150	0.098
G.B.	0.83	0.164	0.100	0.72	0.169	0.124
D.T	0.82	0.178	0.105	0.72	0.188	0.114

**Table 5:** Pearson correlation matrix between any two features of agro waste, and between any variable and the target variable (adsorption efficiency).

Attributes	Adsorption Efficiency	Biochar pH	C_0_	Particle Size	Pore Volume	Surface Area
Adsorption efficiency	1	−0.558	0.739	0.084	0.666	0.690
Biochar pH	−0.558	1	−0.424	0.102	−0.5401	−0.252
C_0_	0.739	−0.424	1	0.095	0.546	0.664
Particle size	0.084	0.102	0.095	1	0.154	0.277
Pore volume	0.666	−0.540	0.546	0.154	1	0.669
Surface area	0.690	−0.252	0.664	0.277	0.669	1

**Table 6:** Importance of variables based on RF model.

Attribute	RF
C_0_	0.347
Pore volume	0.287
Surface area	0.131
Agro-waste pH	0.056
Particle size	0.033

**Table 7:** The optimization process of RF models with error rate.

No.	Number of Trees	Maximal Depth	Error Rate
1	20.0	2.0	0.672
2	60.0	2.0	0.671
3	100.0	2.0	0.673
4	140.0	2.0	0.679
5	20.0	4.0	0.405
6	60.0	4.0	0.392
7	100.0	4.0	0.398
8	140.0	4.0	0.402
**9**	**20.0**	**7.0**	**0.351**
10	60.0	7.0	0.348
11	100.0	7.0	0.350
12	140.0	7.0	0.347

**Table 8:** The predicted importance of variables on the adsorption efficiency.

Attribute	Optimum Values (Normalized)	Optimum Values (Actual Value)	Maximum Adsorption	Maximum Adsorption (Actual Value)
C_0_ (mg/L)	0.357	355	0.81	
Pore volume (cm^3^/g)	0.889	1.027		
Surface area (m^2^/g)	0.863	2106		1813
Agro-waste pH	0.272	2.53		
Particle size (mm)	0.112	0.013		

## Supplementary Materials

The following are available online at https://www.mdpi.com/article/10.3390/nano11102734/s1. Table S1. A Study on Machine Learning Methods’ Application for Dye Adsorption Prediction onto Agricultural Waste Activated Carbon.
