# Supplementary material for: A Study on Machine Learning Methods’ Application for Dye Adsorption Prediction onto Agricultural Waste Activated Carbon
Source: Nanomaterials (Basel). 2021 Oct 15;11(10):2734. doi: 10.3390/nano11102734 (PMC8540925; doi:10.3390/nano11102734)
Supplement: Supplementary file 1 [file nanomaterials-11-02734-s001.zip › nanomaterials-1380363-supplementary.pdf]

Supplementary Material

A Study on Machine Learning Methods' Application for Dye Adsorption Prediction onto Agricultural Waste Activated Carbon

Seyedehmaryam Moosavi [1,*], Otilia Manta [2,3], Yaser A. El-Badry [4], Enas E. Hussein [5], Zeinhom M. El-Bahy [6], Noor fariza Binti Mohd Fawzi [7], Jaunius Urbonavičius [1] and Seyed Mohammad Hossein Moosavi [8]

[1] Department of Chemistry and Bioengineering, Vilnius Gediminas Technical University, 10223 Vilnius, Lithuania; jaunius.urbonavicius@vilniustech.lt
[2] Romanian Academy, Center for Financial and Monetary Research "Victor Slavescu", 050711 Bucharest, Romania; otilia.manta@rgic.ro
[3] Research Department, Romanian-American University, 012101 Bucharest, Romania
[4] Chemistry Department, Faculty of Science, Taif University, khurma, P.O. Box 11099, Taif 21944, Saudi Arabia; y.elbadry@tu.edu.sa
[5] National Water research centre, P.O box 74, Shubra EI-kheima 13411, Egypt; enas_el-sayed@nwrc.gov.eg
[6] Chemistry Department Faculty of Science, Al-Azhar University, Cairo 11884 , Egypt; zeinelbahy@azhar.edu.eg
[7] Nanotechnology & Catalysis Research Centre (NANOCAT), Institute for Advanced Studies (IAS), University for Malaya (UM), Kuala Lumpur, Malaysia; farizafawzi@um.edu.my
[8] Faculty of Engineering, Centre for Transportation Research (CTR), University of Malaya (UM), Kuala Lumpur 50603, Malaysia; mh.moosavi65@gmail.com
* Correspondence: m.moosavi1987@gmail.com

Table S1

		**Agro-waste characteristic**					**Adsorption condition**					
	**Adsorbents**	**Pyrolysis temperature (°C)**	**Agro-waste pH** (pHH2O)	**Particle size (mm)**	**Surface area (m^2/g)**	**Pore volume (cm3/g)**	**Adsorption temperature (T) (°C)**	**Adsorption pH** (pHsol)	**Dyes type**	**C0 (mg/L)**	**Adsorption Efficiency (mg/g)**	**Reference**
1	CS	500	7	0.3	878	0.492	25	7	RD	4	818	1
2	CS	500	7	0.3	878	0.492	25	7	RD	190	1051	1
3	CS	500	7	0.3	878	0.492	25	7	RD	380	1106	1
4	CS	500	7	0.3	878	0.492	25	7	RD	40	970	1
5	CS	500	7	0.3	878	0.492	25	7	YD	1.911	563.06	1
6	CS	500	7	0.3	878	0.492	25	7	YD	13.38	740.693	1
7	CS	500	7	0.3	878	0.492	25	7	YD	59.24	908.472	1
8	CS	500	7	0.3	878	0.492	25	7	YD	118.5	1056.07	1
9	CS	500	7	0.3	878	0.492	25	7	YD	215.9	1117.77	1

10	CS	500	7	0.3	878	0.492	25	7	YD	307.6	1169.27	1
11	CS	500	7	0.3	878	0.492	25	7	YD	401.3	1190.35	1
12	CS	500	7	0.3	878	0.492	25	7	YD	498.7	1216.54	1
13	CS	500	7	0.3	878	0.492	25	7	YD	569.4	1237.42	1
14	AC600	450	4	0.71	1865	0.9191	25	7	MB	100	397	2
15	AC600	450	4	0.71	1865	0.9191	25	7	MB	200	795	2
16	AC600	450	4	0.71	1865	0.9191	25	7	MB	300	1192	2
17	AC600	450	4	0.71	1865	0.9191	25	7	MB	400	1971	2
18	AC600	450	4	0.71	1865	0.9191	25	7	MB	400	1564	2
19	AC600	450	4	0.71	1865	0.9191	25	7	MB	500	1928	2
20	AC600	450	4	0.71	1865	0.9191	25	7	MB	600	1953	2
21	AC600	450	4	0.71	1865	0.9191	25	7	MB	700	1906	2
22	AC600	450	4	0.71	1865	0.9191	25	7	MB	800	1877	2
23	AC700	450	4	0.78	2015	1.0757	25	7	C-Red	400	2235	2
24	AC600	450	4	0.71	1865	0.9191	25	7	MB	900	1972	2
25	AC700	450	4	0.78	2015	1.0757	25	7	CCR	400	2091	2
26	AC700	450	4	0.78	2015	1.0757	25	7	CCB	400	1594	2
27	AC700	450	4	0.78	2015	1.0757	25	7	CCY	400	1128	2
28	AC700	450	4	0.78	2015	1.0757	25	7	CBlue	400	2235	2
29	AC700	450	4	0.78	2015	1.0757	25	7	C-Yellow	400	2091	2
30	AC700	450	4	0.78	2015	1.0757	25	7	MB	300	1192	2
31	AC700	450	4	0.78	2015	1.0757	25	7	MB	200	795	2
32	AC700	450	4	0.78	2015	1.0757	25	7	MB	400	2235	2
33	AC700	450	4	0.78	2015	1.0757	25	7	MB	400	1610	2
34	AC700	450	4	0.78	2015	1.0757	25	2	MB	500	1470	2
35	AC700	450	4	0.78	2015	1.0757	25	4	MB	500	1691	2
36	AC700	450	4	0.78	2015	1.0757	25	6	MB	500	1813	2
37	AC700	450	4	0.78	2015	1.0757	25	8	MB	500	1989	2
38	AC700	450	4	0.78	2015	1.0757	25	10	MB	500	1989	2
39	AC700	450	4	0.78	2015	1.0757	25	7	MB	500	1993	2
40	AC700	450	4	0.78	2015	1.0757	25	7	MB	600	2172	2
41	AC700	450	4	0.78	2015	1.0757	25	7	MB	700	2184	2
42	AC700	450	4	0.78	2015	1.0757	25	7	MB	800	2185	2

43	AC700	450	4	0.78	2015	1.0757	25	7	MB	900	2239	2
44	AC700	450	4	0.78	2015	1.0757	25	7	MB	100	397	2
45	AC800	450	4	0.75	1999	1.1551	25	7	MB	100	397	2
46	AC900	450	4	0.74	1814	1.1587	25	7	MB	100	397	2
47	AC800	450	4	0.75	1999	1.1551	25	7	MB	200	795	2
48	AC800	450	4	0.75	1999	1.1551	25	7	MB	300	1192	2
49	AC800	450	4	0.75	1999	1.1551	25	7	MB	400	1548	2
50	AC800	450	4	0.75	1999	1.1551	25	7	MB	500	1750	2
51	AC800	450	4	0.75	1999	1.1551	25	7	MB	600	1899	2
52	AC800	450	4	0.75	1999	1.1551	25	7	MB	700	1995	2
53	AC800	450	4	0.75	1999	1.1551	25	7	MB	800	2031	2
54	AC800	450	4	0.75	1999	1.1551	25	7	MB	900	1014	2
55	AC900	450	4	0.74	1814	1.1587	25	7	MB	200	795	2
56	AC900	450	4	0.74	1814	1.1587	25	7	MB	300	1192	2
57	AC900	450	4	0.74	1814	1.1587	25	7	MB	400	1548	2
58	AC900	450	4	0.74	1814	1.1587	25	7	MB	500	1697	2
59	AC900	450	4	0.74	1814	1.1587	25	7	MB	600	1793	2
60	AC900	450	4	0.74	1814	1.1587	25	7	MB	700	1752	2
61	AC900	450	4	0.74	1814	1.1587	25	7	MB	800	1806	2
62	AC900	450	4	0.74	1814	1.1587	25	7	MB	900	1729	2
63	CMCAC	800	7	7.5	2430	0.39	45	6	MV	50	514.286	2
64	CMCAC	800	7	7.5	2430	0.39	45	6	MV	100	678.571	2
65	CMCAC	800	7	7.5	2430	0.39	45	6	MV	200	828.571	2
66	CMCAC	800	7	7.5	2430	0.39	45	6	MV	300	892.857	2
67	CMCAC	800	7	7.5	2430	0.39	45	6	MV	400	1007.14	2
68	CMCAC	800	7	7.5	2430	0.39	45	6	MV	500	1071.43	2
69	CMCAC	800	7	7.5	2430	0.39	45	6	MV	600	1142.86	2
70	CMCAC	800	7	7.5	2430	0.39	45	6	MV	700	1192.86	2
71	CMCAC	800	7	7.5	2430	0.39	35	6	MV	50	492.857	2
72	CMCAC	800	7	7.5	2430	0.39	35	6	MV	100	707.143	2
73	CMCAC	800	7	7.5	2430	0.39	35	6	MV	200	850	2
74	CMCAC	800	7	7.5	2430	0.39	35	6	MV	300	992.857	2
75	CMCAC	800	7	7.5	2430	0.39	35	6	MV	400	1107.14	2

76	CMCAC	800	7	7.5	2430	0.39	35	6	MV	500	1171.43	2
77	CMCAC	800	7	7.5	2430	0.39	35	6	MV	600	1178.57	2
78	CMCAC	800	7	7.5	2430	0.39	35	6	MV	700	1200	2
79	CMCAC	800	7	0.15	2430	0.39	25	6	MV	50	535.714	3
80	CMCAC	800	7	0.15	2430	0.39	25	6	MV	100	721.429	3
81	CMCAC	800	7	0.15	2430	0.39	25	6	MV	200	850	3
82	CMCAC	800	7	0.15	2430	0.39	25	6	MV	300	1057.14	3
83	CMCAC	800	7	0.15	2430	0.39	25	6	MV	400	1121.43	3
84	CMCAC	800	7	0.15	2430	0.39	25	6	MV	500	1142.86	3
85	CMCAC	800	7	0.15	2430	0.39	25	6	MV	600	1171.43	3
86	CMCAC	800	7	0.15	2430	0.39	25	6	MV	700	1171.43	3
87	CMCAC	800	7	0.15	2430	0.39	45	7	CR	13.27	875.41	3
88	CMCAC	800	7	0.15	2430	0.39	45	7	CR	89.57	1091.8	3
89	CMCAC	800	7	0.15	2430	0.39	45	7	CR	172.5	1259.02	3
90	CMCAC	800	7	0.15	2430	0.39	45	7	CR	272	1308.2	3
91	CMCAC	800	7	0.15	2430	0.39	45	7	CR	364.9	1347.54	3
92	CMCAC	800	7	0.15	2430	0.39	45	7	CR	464.5	1386.89	3
93	CMCAC	800	7	0.15	2430	0.39	45	7	CR	557.3	1445.9	3
94	CMCAC	800	7	0.15	2430	0.39	45	7	CR	656.9	1495.08	3
95	CMCAC	800	7	0.15	2430	0.39	35	7	CR	13.27	885.246	3
96	CMCAC	800	7	0.15	2430	0.39	35	7	CR	86.26	1131.15	3
97	CMCAC	800	7	0.15	2430	0.39	35	7	CR	165.9	1327.87	3
98	CMCAC	800	7	0.15	2430	0.39	35	7	CR	258.8	1416.39	3
99	CMCAC	800	7	0.15	2430	0.39	35	7	CR	345	1554.1	3
100	CMCAC	800	7	0.15	2430	0.39	35	7	CR	441.2	1573.77	3
101	CMCAC	800	7	0.15	2430	0.39	35	7	CR	540.8	1622.95	3
102	CMCAC	800	7	0.15	2430	0.39	35	7	CR	637	1622.95	3
103	CMCAC	800	7	0.15	2430	0.39	25	7	CR	20	777.049	3
104	CMCAC	800	7	0.15	2430	0.39	25	7	CR	99.53	993.443	3
105	CMCAC	800	7	0.15	2430	0.39	25	7	CR	175.8	1268.85	3
106	CMCAC	800	7	0.15	2430	0.39	25	7	CR	255.5	1445.9	3
107	CMCAC	800	7	0.15	2430	0.39	25	7	CR	348.3	1504.92	3
108	CMCAC	800	7	0.15	2430	0.39	25	7	CR	441.2	1593.44	3

109	CMCAC	800	7	0.15	2430	0.39	25	7	CR	540.8	1603.28	3
110	CMCAC	800	7	0.15	2430	0.39	25	7	CR	637	1642.62	3
111	CMCAC	800	7	0.15	2430	0.39	45	7	AR	100	292.453	3
112	CMCAC	800	7	0.15	2430	0.39	45	7	AR	200	371.096	3
113	CMCAC	800	7	0.15	2430	0.39	45	7	AR	300	395.567	3
114	CMCAC	800	7	0.15	2430	0.39	45	7	AR	400	426.062	3
115	CMCAC	800	7	0.15	2430	0.39	45	7	AR	500	450.518	3
116	CMCAC	800	7	0.15	2430	0.39	45	7	AR	600	456.917	3
117	CMCAC	800	7	0.15	2430	0.39	45	7	AR	700	472.352	3
118	CMCAC	800	7	0.15	2430	0.39	45	7	AR	800	499.82	3
119	CMCAC	800	7	0.15	2430	0.39	35	7	AR	100	322.544	3
120	CMCAC	800	7	0.15	2430	0.39	35	7	AR	200	359.048	3
121	CMCAC	800	7	0.15	2430	0.39	35	7	AR	300	386.516	3
122	CMCAC	800	7	0.15	2430	0.39	35	7	AR	400	401.966	3
123	CMCAC	800	7	0.15	2430	0.39	35	7	AR	500	420.398	3
124	CMCAC	800	7	0.15	2430	0.39	35	7	AR	600	426.797	3
125	CMCAC	800	7	0.15	2430	0.39	35	7	AR	700	433.195	3
126	CMCAC	800	7	0.15	2430	0.39	35	7	AR	800	442.606	3
127	CMCAC	800	7	0.15	2430	0.39	25	7	AR	100	66.6097	3
128	CMCAC	800	7	0.15	2430	0.39	25	7	AR	200	103.129	3
129	CMCAC	800	7	0.15	2430	0.39	25	7	AR	300	130.612	3
130	CMCAC	800	7	0.15	2430	0.39	25	7	AR	400	151.936	3
131	CMCAC	800	7	0.15	2430	0.39	25	7	AR	500	167.491	3
132	CMCAC	800	7	0.15	2430	0.39	25	7	AR	600	176.902	3
133	CMCAC	800	7	0.15	2430	0.39	25	7	AR	700	168.24	3
134	CMCAC	800	7	0.15	2430	0.39	25	7	AR	800	177.666	3
135	CS-AC	400	7	2.17	2197	1.192	20	7	GR	94	971.739	4
136	CS-AC	400	7	2.17	2197	1.192	20	7	GR	147	1428.26	4
137	CS-AC	400	7	2.17	2197	1.192	20	7	GR	300	1447.83	4
138	CS-AC	400	7	2.17	2197	1.192	20	7	GR	500	1532.61	4
139	CS-AC	400	7	2.17	2197	1.192	20	7	GR	900	1623.91	4
140	CS-AC	400	7	2.17	2197	1.192	20	7	GR	1000	1480.43	4
141	CS-AC	400	7	2.17	2197	1.192	20	7	GR	94	971.739	4

142	CS-AC	400	7	2.17	2197	1.192	20	7	GR	110	1506.52	4
143	CS-AC	400	7	2.17	2197	1.192	20	7	GR	240	1584.78	4
144	CS-AC	400	7	2.17	2197	1.192	20	7	GR	400	1773.91	4
145	CS-AC	400	7	2.17	2197	1.192	20	7	GR	800	1819.57	4
146	CS-AC	400	7	2.17	2197	1.192	20	7	GR	1000	1636.96	4
147	CS-AC	400	7	2.17	2197	1.192	20	7	GR	97	71.7391	4
148	CS-AC	400	7	2.17	2197	1.192	20	7	GR	107	97.8261	4
149	CS-AC	400	7	2.17	2197	1.192	20	7	GR	120	104.348	4
150	CS-AC	400	7	2.17	2197	1.192	20	7	GR	137	97.8261	4
151	CS-AC	400	7	2.17	2197	1.192	20	7	GR	167	97.8261	4
152	CS-AC	400	7	2.17	2197	1.192	20	7	GR	96.94	71.7391	4
153	CS-AC	400	7	2.17	2197	1.192	20	7	GR	103.6	91.3043	4
154	CS-AC	400	7	2.17	2197	1.192	20	7	GR	113.6	110.87	4
155	CS-AC	400	7	2.17	2197	1.192	20	7	GR	123.7	123.913	4
156	CS-AC	400	7	2.17	2197	1.192	20	7	GR	150.4	110.87	4
157	CS-AC	400	7	2.17	2197	1.192	20	7	GR	500	936.759	4
158	CS-AC	400	7	2.17	2197	1.192	20	7	GR	500	992.095	4
159	CS-AC	400	7	2.17	2197	1.192	20	7	GR	500	173.913	4
160	CS-AC	400	7	2.17	2197	1.192	20	7	GR	500	221.344	4
161	TSAC	650	7	0.5	222	0.1832	25	6.5	RB5	50	5	5
162	TSAC	650	7	0.5	222	0.1832	25	6.2	MB	50	3.5	5
163	TSAC	650	7	0.5	222	0.1832	25	6.5	RB5	50	11.98	5
164	TSAC	650	7	0.5	222	0.1832	25	6.2	MB	50	13.27	5
165	TSAC	650	7	0.5	222	0.1832	25	6.2	MB	10	0.65625	5
166	TSAC	650	7	0.5	222	0.1832	25	6.2	MB	50	3.625	5
167	TSAC	650	7	0.5	222	0.1832	25	6.2	MB	100	4.46875	5
168	TSAC	650	7	0.5	222	0.1832	25	6.2	MB	150	4.9375	5
169	TSAC	650	7	0.5	222	0.1832	25	6.2	MB	200	5.3125	5
170	TSAC	650	7	0.5	222	0.1832	25	6.5	RB5	10	1.03125	5
171	TSAC	650	7	0.5	222	0.1832	25	6.5	RB5	50	5	5
172	TSAC	650	7	0.5	222	0.1832	25	6.5	RB5	100	5.53125	5
173	TSAC	650	7	0.5	222	0.1832	25	6.5	RB5	150	6.125	5
174	TSAC	650	7	0.5	222	0.1832	25	6.5	RB5	200	6.4375	5

175	TSAC	650	7	0.5	222	0.1832	30	6.5	RB5	10	0.671642	5
176	TSAC	650	7	0.5	222	0.1832	30	6.5	RB5	50	3.58209	5
177	TSAC	650	7	0.5	222	0.1832	30	6.5	RB5	100	5.52239	5
178	TSAC	650	7	0.5	222	0.1832	30	6.5	RB5	150	6.1194	5
179	TSAC	650	7	0.5	222	0.1832	30	6.5	RB5	200	6.41791	5
180	TSAC	650	7	0.5	222	0.1832	50	6.5	RB5	10	0.970149	5
181	TSAC	650	7	0.5	222	0.1832	50	6.5	RB5	50	4.92537	5
182	TSAC	650	7	0.5	222	0.1832	50	6.5	RB5	100	9.70149	5
183	TSAC	650	7	0.5	222	0.1832	50	6.5	RB5	150	9.55224	5
184	TSAC	650	7	0.5	222	0.1832	50	6.5	RB5	200	9.62687	5
185	TSAC	650	7	0.5	222	0.1832	60	6.5	RB5	10	0.895522	5
186	TSAC	650	7	0.5	222	0.1832	60	6.5	RB5	50	5.07463	5
187	TSAC	650	7	0.5	222	0.1832	60	6.5	RB5	100	10.0746	5
188	TSAC	650	7	0.5	222	0.1832	60	6.5	RB5	150	15.3731	5
189	TSAC	650	7	0.5	222	0.1832	60	6.5	RB5	200	17.2388	5
190	TSAC	650	7	0.5	222	0.1832	50	6.2	MB	10	0.820896	5
191	TSAC	650	7	0.5	222	0.1832	50	6.2	MB	50	3.65672	5
192	TSAC	650	7	0.5	222	0.1832	50	6.2	MB	100	6.86567	5
193	TSAC	650	7	0.5	222	0.1832	50	6.2	MB	150	8.20896	5
194	TSAC	650	7	0.5	222	0.1832	50	6.2	MB	200	8.80597	5
195	TSAC	650	7	0.5	222	0.1832	60	6.2	MB	10	1.04478	5
196	TSAC	650	7	0.5	222	0.1832	60	6.2	MB	50	4.1791	5
197	TSAC	650	7	0.5	222	0.1832	60	6.2	MB	100	7.16418	5
198	TSAC	650	7	0.5	222	0.1832	60	6.2	MB	150	8.95522	5
199	TSAC	650	7	0.5	222	0.1832	60	6.2	MB	200	9.25373	5
200	MC350	350	7	0.48	49	0.033	25	7	MB	10	1.9661	6
201	MC350	350	7	0.48	49	0.033	25	7	MB	20	5.15254	6
202	MC350	350	7	0.48	49	0.033	25	7	MB	30	8.27119	6
203	MC350	350	7	0.48	49	0.033	25	7	MB	40	12.6102	6
204	MC400	400	7	0.57	72	0.053	25	7	MB	10	2.37288	6
205	MC400	400	7	0.57	72	0.053	25	7	MB	20	5.22034	6
206	MC400	400	7	0.57	72	0.053	25	7	MB	30	8.94915	6
207	MC400	400	7	0.57	72	0.053	25	7	MB	40	12.8136	6

208	MC450	450	7	0.66	81	0.087	25	7	MB	10	2	6
209	MC450	450	7	0.66	81	0.087	25	7	MB	20	5	6
210	MC450	450	7	0.66	81	0.087	25	7	MB	30	8	6
211	MC450	450	7	0.66	81	0.087	25	7	MB	40	12.1	6
212	MC500	500	7	0.66	116	0.134	25	7	MB	10	2.44068	6
213	MC500	500	7	0.66	116	0.134	25	7	MB	20	5.89831	6
214	MC500	500	7	0.66	116	0.134	25	7	MB	30	9.76271	6
215	MC500	500	7	0.66	116	0.134	25	7	MB	40	13.8305	6
216	MC550	550	7	0.48	116	0.183	25	7	MB	10	2.98305	6
217	MC550	550	7	0.48	116	0.183	25	7	MB	20	6.37288	6
218	MC550	550	7	0.48	116	0.183	25	7	MB	30	10.1017	6
219	MC550	550	7	0.48	116	0.183	25	7	MB	40	14.9153	6
220	MC600	600	7	0.75	49	0.033	25	7	MB	10	2.37288	6
221	MC600	600	7	0.75	49	0.033	25	7	MB	20	5.69492	6
222	MC600	600	7	0.75	49	0.033	25	7	MB	30	8.94915	6
223	MC600	600	7	0.75	49	0.033	25	7	MB	40	12.9492	6
224	MC30	550	7	0.48	116	0.183	25	7	MB	8	5	6
225	MC30	550	7	0.48	116	0.183	25	7	MB	12	9.0566	6
226	MC30	550	7	0.48	116	0.183	25	7	MB	18	11.0377	6
227	MC30	550	7	0.48	116	0.183	25	7	MB	28	13.0189	6
228	MC30	550	7	0.48	116	0.183	25	7	MB	36	14.5283	6
229	MC60	550	7	0.48	116	0.183	25	7	MB	8	5.9434	6
230	MC60	550	7	0.48	116	0.183	25	7	MB	12	10.4717	6
231	MC60	550	7	0.48	116	0.183	25	7	MB	18	12.3585	6
232	MC60	550	7	0.48	116	0.183	25	7	MB	28	13.6792	6
233	MC60	550	7	0.48	116	0.183	25	7	MB	36	16.5094	6
234	MC120	550	7	0.48	116	0.183	25	7	MB	8	5.84906	6
235	MC120	550	7	0.48	116	0.183	25	7	MB	12	10.6604	6
236	MC120	550	7	0.48	116	0.183	25	7	MB	18	13.2075	6
237	MC120	550	7	0.48	116	0.183	25	7	MB	28	14.2453	6
238	MC120	550	7	0.48	116	0.183	25	7	MB	36	15.9434	6
239	MC90	550	7	0.48	116	0.183	25	7	MB	8	7.16981	6
240	MC90	550	7	0.48	116	0.183	25	7	MB	12	9.90566	6

241	MC90	550	7	0.48	116	0.183	25	7	MB	18	13.9623	6
242	MC90	550	7	0.48	116	0.183	25	7	MB	28	15	6
243	MC90	550	7	0.48	116	0.183	25	7	MB	36	17.0755	6
244	MC0.75	550	7	0.48	116	0.183	25	7	MB	8	4.09756	6
245	MC0.75	550	7	0.48	116	0.183	25	7	MB	12	10.1463	6
246	MC0.75	550	7	0.48	116	0.183	25	7	MB	18	9.95122	6
247	MC0.75	550	7	0.48	116	0.183	25	7	MB	28	10.9268	6
248	MC0.75	550	7	0.48	116	0.183	25	7	MB	36	12	6
249	MC0.659	550	7	0.48	116	0.183	25	7	MB	8	6.43902	6
250	MC0.659	550	7	0.48	116	0.183	25	7	MB	12	11.7073	6
251	MC0.659	550	7	0.48	116	0.183	25	7	MB	18	12.9756	6
252	MC0.659	550	7	0.48	116	0.183	25	7	MB	28	15.3171	6
253	MC0.659	550	7	0.48	116	0.183	25	7	MB	36	15.8049	6
254	MC0.569	550	7	0.48	116	0.183	25	7	MB	8	6.63415	6
255	MC0.569	550	7	0.48	116	0.183	25	7	MB	12	12.0976	6
256	MC0.569	550	7	0.48	116	0.183	25	7	MB	18	14.0488	6
257	MC0.569	550	7	0.48	116	0.183	25	7	MB	28	16.0976	6
258	MC0.569	550	7	0.48	116	0.183	25	7	MB	36	17.3659	6
259	MC0.478	550	7	0.48	116	0.183	25	7	MB	8	7.21951	6
260	MC0.478	550	7	0.48	116	0.183	25	7	MB	12	13.0732	6
261	MC0.478	550	7	0.48	116	0.183	25	7	MB	18	15.2195	6
262	MC0.478	550	7	0.48	116	0.183	25	7	MB	28	16.9756	6
263	MC0.478	550	7	0.48	116	0.183	25	7	MB	36	18.1463	6
264	MC20/1	550	7	0.48	116	0.183	25	7	MB	8	3.94089	6
265	MC20/1	550	7	0.48	116	0.183	25	7	MB	12	7.9803	6
266	MC20/1	550	7	0.48	116	0.183	25	7	MB	18	10.0493	6
267	MC20/1	550	7	0.48	116	0.183	25	7	MB	28	8.96552	6
268	MC20/1	550	7	0.48	116	0.183	25	7	MB	36	10.0493	6
269	MC251	550	7	0.48	116	0.183	25	7	MB	8	4.33498	6
270	MC251	550	7	0.48	116	0.183	25	7	MB	12	8.37438	6
271	MC251	550	7	0.48	116	0.183	25	7	MB	18	10.7389	6
272	MC251	550	7	0.48	116	0.183	25	7	MB	28	9.85222	6
273	MC251	550	7	0.48	116	0.183	25	7	MB	36	11.9212	6

274	MC301	550	7	0.48	116	0.183	25	7	MB	8	5.12315	6
275	MC301	550	7	0.48	116	0.183	25	7	MB	12	9.16256	6
276	MC301	550	7	0.48	116	0.183	25	7	MB	18	11.133	6
277	MC301	550	7	0.48	116	0.183	25	7	MB	28	13.0049	6
278	MC301	550	7	0.48	116	0.183	25	7	MB	36	14.9754	6
279	MC351	550	7	0.48	116	0.183	25	7	MB	8	6.99507	6
280	MC351	550	7	0.48	116	0.183	25	7	MB	12	10.936	6
281	MC351	550	7	0.48	116	0.183	25	7	MB	18	13.7931	6
282	MC351	550	7	0.48	116	0.183	25	7	MB	28	14.9754	6
283	MC351	550	7	0.48	116	0.183	25	7	MB	36	18.0296	6
284	MCNaOH10	550	7	0.48	116	0.183	25	7	MB	6	5.12195	6
285	MCNaOH10	550	7	0.48	116	0.183	25	7	MB	9	7.92683	6
286	MCNaOH10	550	7	0.48	116	0.183	25	7	MB	17	10.9756	6
287	MCNaOH10	550	7	0.48	116	0.183	25	7	MB	24	10.8537	6
288	MCNaOH10	550	7	0.48	116	0.183	25	7	MB	30	12.9268	6
289	MCNaOH30	550	7	0.48	116	0.183	25	7	MB	6	6.09756	6
290	MCNaOH30	550	7	0.48	116	0.183	25	7	MB	9	10	6
291	MCNaOH30	550	7	0.48	116	0.183	25	7	MB	17	13.9024	6
292	MCNaOH30	550	7	0.48	116	0.183	25	7	MB	24	13.9024	6
293	MCNaOH30	550	7	0.48	116	0.183	25	7	MB	30	17.9268	6
294	MCNaOH40	550	7	0.48	116	0.183	25	7	MB	6	7.56098	6
295	MCNaOH40	550	7	0.48	116	0.183	25	7	MB	9	11.7073	6
296	MCNaOH40	550	7	0.48	116	0.183	25	7	MB	17	14.1463	6
297	MCNaOH40	550	7	0.48	116	0.183	25	7	MB	24	15	6
298	MCNaOH40	550	7	0.48	116	0.183	25	7	MB	30	20.2439	6
299	MCNaOH50	550	7	0.48	116	0.183	25	7	MB	6	8.29268	6
300	MCNaOH50	550	7	0.48	116	0.183	25	7	MB	9	13.1707	6
301	MCNaOH50	550	7	0.48	116	0.183	25	7	MB	17	16.0976	6
302	MCNaOH50	550	7	0.48	116	0.183	25	7	MB	24	18.0488	6
303	MCNaOH50	550	7	0.48	116	0.183	25	7	MB	30	21.9512	6
304	GSAC-Ce-1	900	4.2	0.2	1258	0.35	25	7	MB	400	199.76	7
305	GSAC	900	4.2	0.2	1102	0.19	25	11.67	MB	400	270.27	7
306	CAC	800	8	1.2	1223	0.72	25	6.8	NR	400	415.023	8

307	CBAC	800	8	1.2	893	0.52	25	6.8	NR	400	105.164	8
308	HAC	800	8	1.2	760	0.47	25	6.8	NR	400	110.798	8
309	CAC	800	8	1.2	1223	0.72	25	6.8	NR	100	97.7376	8
310	CBAC	800	8	1.2	893	0.52	25	6.8	NR	100	73.7557	8
311	HAC	800	8	1.2	760	0.47	25	6.8	NR	100	58.8235	8
312	VAC	650	6.8	5	598	0.37	25	6.8	AM	50	12.3	9
313	VAC	650	6.8	5	598	0.37	25	4.6	AM	100	25.3	9
314	VAC	650	6.8	5	598	0.37	25	4.6	MB	50	12.6	9
315	VAC	650	6.8	5	598	0.37	25	4.6	MB	100	25.1	9
316	SAC	650	6.8	1.18	98.8	0.09	25	4.6	MB	100	19.6	9
317	SAC	650	6.8	1.18	98.8	0.09	25	4.6	MB	10	2.44	9
318	SAC	650	6.8	1.18	98.8	0.09	25	4.6	MB	50	12.3	9
319	SAC	650	6.8	1.18	98.8	0.09	25	4.6	AM	10	2.24	9
320	SAC	650	6.8	1.18	98.8	0.09	25	4.6	AM	50	12.2	9
321	SAC	650	6.8	1.18	98.8	0.09	25	4.6	AM	100	23.6	9
322	SAC	650	6.8	1.18	98.8	0.09	25	4.6	AM	170	12.3188	9
323	VAC	650	6.8	5	598	0.37	25	4.6	AM	170	25	9
324	SAC	650	6.8	1.18	98.8	0.09	25	4.6	MB	170	19.5652	9
325	VAC	650	6.8	5	598	0.37	25	4.6	MB	170	25	9
326	TRAC	550	4	1	121	0.1514	25	10	MB	200	404.7	10
327	TRAC	550	4	1	121	0.1514	25	10	AB25	100	399.58	10
328	TRAC	550	4	1	121	0.1514	25	10	MB	100	151.405	10
329	TRAC	550	4	1	121	0.1514	25	10	AB25	100	153.189	10
330	BGBHAC	400	8	1.94	1710	0.834	21	7	MO	50	99.88	11
331	BGBHAC	400	8	1.94	1710	0.834	21	7	MO	100	199.5	11
332	BGBHAC	400	8	1.94	1710	0.834	21	7	MO	150	298.2	11
333	BGBHAC	400	8	1.94	1710	0.834	21	7	MO	200	392.8	11
334	BGBHAC	400	8	1.94	1710	0.834	21	7	MB	50	98.9772	11
335	BGBHAC	400	8	1.94	1710	0.834	21	7	MB	100	198.6	11
336	BGBHAC	400	8	1.94	1710	0.834	21	7	MB	150	295.9	11
337	BGBHAC	400	8	1.94	1710	0.834	21	7	MB	200	390.3	11
338	BGBHAC	400	8	1.94	1710	0.834	21	7	MB	100	469	11
339	BGBHAC	400	8	1.94	1710	0.834	21	7	MO	100	418	11

346	WSAC	700	7	4.4	1145	0.53	25	7.3	Rhd B	50	123.46	13
347	WSAC	700	7	4.4	1145	0.53	25	7.3	Rhd B	25	29.3671	13
348	WSAC	700	7	4.4	1145	0.53	25	7.3	Rhd B	50	60.2532	13
349	WSAC	700	7	4.4	1145	0.53	25	7.3	Rhd B	75	83.038	13
350	WSAC	700	7	4.4	1145	0.53	25	7.3	Rhd B	100	104.81	13

1. Lu P-J, Lin H-C, Yu W-T, Chern J-M. Chemical regeneration of activated carbon used for dye adsorption. Journal of the Taiwan institute of chemical engineers. 2011;42(2):305-11.
2. Li L, Wu M, Song C, Liu L, Gong W, Ding Y, et al. Efficient removal of cationic dyes via activated carbon with ultrahigh specific surface derived from vinasse wastes. Bioresource Technology. 2020:124540.
3. Wang H, Li Z, Yahyaoui S, Hanafy H, Seliem MK, Bonilla-Petriciolet A, et al. Effective adsorption of dyes on an activated carbon prepared from carboxymethyl cellulose: Experiments, characterization and advanced modelling. Chemical Engineering Journal. 2020:128116.
4. Gao Y, Xu S, Yue Q, Wu Y, Gao B. Chemical preparation of crab shell-based activated carbon with superior adsorption performance for dye removal from wastewater. Journal of the Taiwan Institute of Chemical Engineers. 2016;61:327-35.
5. Wong S, Yac'cob NAN, Ngadi N, Hassan O, Inuwa IM. From pollutant to solution of wastewater pollution: Synthesis of activated carbon from textile sludge for dye adsorption. Chinese Journal of Chemical Engineering. 2018;26(4):870-8.
6. Shokry H, Elkady M, Hamad H. Nano activated carbon from industrial mine coal as adsorbents for removal of dye from simulated textile wastewater: Operational parameters and mechanism study. Journal of Materials Research and Technology. 2019;8(5):4477-88.
7. Alshabib M, Oluwadamilare MA, Tanimu A, Abdulazeez I, Alhooshani K, Ganiyu SA. Experimental and DFT investigation of ceria-nanocomposite decorated AC derived from groundnut shell for efficient removal of methylene-blue from wastewater effluent. Applied Surface Science. 2021;536:147749.
8. Mei S, Gu J, Ma T, Li X, Hu Y, Li W, et al. N-doped activated carbon from used dyeing wastewater adsorbent as a metal-free catalyst for acetylene hydrochlorination. Chemical Engineering Journal. 2019;371:118-29.
9. Ravenni G, Cafaggi G, Sárossy Z, Nielsen KR, Ahrenfeldt J, Henriksen U. Waste chars from wood gasification and wastewater sludge pyrolysis compared to commercial activated carbon for the removal of cationic and anionic dyes from aqueous solution. Bioresource Technology Reports. 2020;10:100421.
10. Archin S, Sharifi SH, Asadpour G. Optimization and modeling of simultaneous ultrasound-assisted adsorption of binary dyes using activated carbon from tobacco residues: response surface methodology. Journal of Cleaner Production. 2019;239:118136.
11. Gupta K, Gupta D, Khatri OP. Graphene-like porous carbon nanostructure from Bengal gram bean husk and its application for fast and efficient adsorption of organic dyes. Applied Surface Science. 2019;476:647-57.
12. Rani KM, Palanisamy P, Gayathri S, Tamilselvi S. Adsorptive removal of basic violet dye from aqueous solution by activated carbon prepared from tea dust material. The International Journal of Innovative Research in Science, Engineering and Technology. 2015;4(8):6845-53.
13. Xiao W, Garba ZN, Sun S, Lawan I, Wang L, Lin M, et al. Preparation and evaluation of an effective activated carbon from white sugar for the adsorption of rhodamine B dye. Journal of Cleaner Production. 2020;253:119989.
14. Mudyawabikwa B, Mungondori HH, Tichagwa L, Katwire DM. Methylene blue removal using a low-cost activated carbon adsorbent from tobacco stems: kinetic and equilibrium studies. Water Science and Technology. 2017;75(10):2390-402.
